# Application of the 2016 diagnostic approach for autoimmune encephalitis from *Lancet Neurology* to Chinese patients

**DOI:** 10.1186/s12883-017-0974-3

**Published:** 2017-11-06

**Authors:** Lin Li, Lin Sun, Rong Du, Yuanchu Zheng, Feifei Dai, Qiuying Ma, Jiawei Wang

**Affiliations:** 1 Department of Neurology, Beijing Tongren Hospital, Capital Medical University, No.1 Dongjiaominxiang, Dongcheng District, Beijing, 100730 China; 2 Medical Research Center, Beijing Tongren Hospital, Capital Medical University, No.1 Dongjiaominxiang, Dongcheng District, Beijing, 100730 China

**Keywords:** Autoimmune encephalitis, Diagnostic approach, Evaluation, Sensitivity, Specificity

## Abstract

**Background:**

A unified clinical approach to diagnose autoimmune encephalitis was published in *Lancet Neurology* in 2016. Purpose of our study is to examine the feasibility and reasonability of the 2016 “A clinical approach to diagnosis of autoimmune encephalitis” in China with a retrospective study.

**Methods:**

We retrospectively collected 95 cases of autoimmune encephalitis and non autoimmune encephalitis cases with detailed clinical data from Beijing Tongren Hospital and the China National Knowledge Infrastructure (CNKI). All cases were analysed stepwise according to the approach in *Lancet Neurology* to compare the new diagnosis with the final clinical diagnosis.

**Results:**

The disease course of these 95 cases ranged from 2 to 540 days. Initial symptoms include fever, headache, seizure, mental and behavioral disorders, memory deterioration and illusion. Based on symptoms and signs when the patient came to the hospital, the sensitivity and specificity of criteria were as follows: possible autoimmune encephalitis (pAE) 84% and 94%, definite autoimmune limbic encephalitis (dALE) 38% and 96%, probable anti-N-methyl-D-aspartate receptor encephalitis (prNMDARE) 49% and 98%. The sensitivities of the above three criteria and the specificity of pAE were low during early disease stage, while the specificities of dALE and prNMDAER remained relatively high in different time periods.

**Conclusions:**

This new autoimmune encephalitis diagnostic approach can recognize possible autoimmune encephalitis. The chances of a case being autoimmune-mediated following classification as autoimmune encephalitis with the new criteria are high. The flowchart is recommended to use as a whole. At the early disease stage, criteria with low sensitivity and high specificity, such as dALE and prNMDARE, lead most cases to enter subsequent diagnosis steps, namely autoantibody detection in the flowchart. Final diagnoses can only be made by autoantibody tests. These factors may make it challenging for clinicians to make diagnosis promptly and to begin immune-modulating therapy immediately. Moreover, the criteria for patients with paraneoplastic syndromes (PNSs) should be considered to avoid diagnosis omission. For Chinese patients, a multi-centre, prospective study on the clinical manifestations, laboratory diagnostic technology, therapy, and prognosis is greatly needed.

**Electronic supplementary material:**

The online version of this article (10.1186/s12883-017-0974-3) contains supplementary material, which is available to authorized users.

## Background

Autoimmune encephalitis has attracted much attention within the last decade. The discovery of autoimmune antibodies targeting proteins in the brain has been applied to the diagnosis of encephalitis, psychiatric disease, seizures, dyskinesia and cognitive impairment.

As early as the 1960s, limbic encephalitis was described as a subacute onset of episodic memory loss, confusion and agitation. Neurologists proposed that this disease was typically associated with neoplasms. To some extent, antibodies against nuclear and cytoplasmic proteins, such as Hu, Ma, and Ri, represent specific malignancies. These antibodies are not directly pathogenic. However, novel autoantibodies against cell surface or synaptic proteins can alter the function, structure, or density of neuronal antigens, causing nervous system dysfunction. Because neuronal cell surface antigenic epitopes are extracellular rather than intracellular [1–4], these novel antibodies are also known as ‘neuronal surface antibodies’ (NSAbs) [5]. Clinical manifestations of autoimmune encephalitis can be serious and may cause death. Research studies on the mechanisms of autoimmune encephalitis have shown that autoantibodies can either induce internalization of antigens or block antigen binding sites, resulting in decreases in other receptors via unknown mechanisms [6]. Previously discovered mechanisms have revealed a reversible process once these antibodies are removed, which may explain the good prognosis of patients with autoimmune encephalitis. Thus, early identification and diagnosis of this disease are very important.

Differential diagnoses between autoimmune encephalitis and other diseases such as infectious encephalitis are difficult at the early stage of the disease, because symptoms are similar among these patients, and even some cases of autoimmune encephalitis can be triggered by herpes simplex encephalitis (HSE) [7–9]. Moreover, Peng et al. reported anti- N-methyl-D-aspartate (anti-NMDA) receptor encephalitis triggered by an *Angiostrongylus cantonesis* infection [10]. Currently, a definite diagnosis of autoimmune encephalitis relies heavily on the detection of autoimmune antibodies. Therefore, a normative diagnostic criterion is greatly needed. Francesc Graus, Angela Vincent, Josep Dalmau and colleagues published “A clinical approach to diagnosis of autoimmune encephalitis” (hereafter referred to as the new approach) in *Lancet Neurology* in 2016 [11]. This approach is based on neurological assessment and conventional tests that are accessible to most clinicians and provide a practical method for early diagnosis. The application of this approach in the clinic has not been reported to date. We analyzed serial clinical and laboratory data that were retrospectively acquired from Beijing Tongren Hospital and the China National Knowledge Infrastructure (CNKI). By applying the new approach for diagnoses of these patients, we aimed to confirm its feasibility, sensitivity, and specificity in Chinese patients, especially during the early disease stage.

## Methods

We retrospectively collected 95 cases, including autoimmune encephalitis and non-autoimmune encephalitis cases. To ensure a balanced distribution of these cases, 49 cases were collected from Beijing Tongren Hospital between January 2012 and June 2016, and the remaining 46 cases were published as “definite autoimmune encephalitis” in CNKI with detailed clinical data from different medical centers. The final clinical diagnosis (reference standard) was made by physicians based on all clinical information, autoantibody tests results (autoanbibody tests methods were cell-based assay, CSF and serum were all included and CSF results were the reference indicator), subsequent course, treatment, prognosis and follow-up. General information, clinical course, symptoms, signs, laboratory test results, image findings and electroencephalography (EEG) results were recorded in detail. Clinicians had no access to information that could identify individual participants and the final clinical diagnoses during or after data collection by elimination of identifying information in advance. Cases with missing data were removed.

We first assumed an unknown autoantibody status and evaluated the autoimmune encephalitis diagnostic approach step-by-step based on the manifestation and laboratory outcomes of each case. Each case was diagnosed by two clinicians using the new approach. Then we assessed the usefulness of the new diagnostic criteria by comparing the new diagnosis with the previous final clinical diagnosis. Additionally, to investigate the diagnostic values of the new criteria in the early stage of the disease, we stratified the disease course into four groups to calculate the diagnosis rate.

We calculated the number of true positives (TP; both diagnoses were autoimmune encephalitis), true negatives (TN; both diagnoses were non-autoimmune encephalitis), false positives (FP; new diagnosis was autoimmune encephalitis and reference standard was non-autoimmune encephalitis), and false negatives (FN; new diagnosis was non-autoimmune encephalitis and reference standard was autoimmune encephalitis) and used the results to determine the following:

1. Sensitivity: the probability of the approach detecting the disease among patients who have the disease (TP/(TP + FN))
2. Specificity: the probability of the approach detecting no disease among patients who do not have the disease (TN/(TN + FP)).
3. Accuracy: the proximity to the true value ((TP + TN)/(TP + TN + FN + FP)).

Chi-square-tests were used to compare rate data between different subgroups, with p<0.05 as threshold for significance. All statistical calculations were carried out with SPSS.

## Results

### General clinical data

We collected 95 cases, including 46 women and 49 men. The median age of disease onset was 34.50 years (range, 9–76 years).The median course when the patient came to hospital was 20.0 days (range, 3–540 days). There were 64 autoimmune encephalitis cases, and 39 cases were diagnosed as anti-NMDA receptor encephalitis, 13 cases as leucine-rich, glioma inactivated 1 (LGI1) Ab(+)-associated limbic encephalitis, 6 cases as gamma-aminobutyric acid-B receptor (GABA_b_R) Ab(+)-associated limbic encephalitis, 1 case as α-amino-3-hydroxy-5-methyl-4-isoxazolepropionic acid receptor (AMPAR) Ab(+)-associated limbic encephalitis, 1 case as Ma_2_ Ab(+)-associated limbic encephalitis, 2 cases as paraneoplastic limbic encephalitis (one case with negative autoantibody, one case with unknown autoantibody status), 1 case as autoimmune limbic encephalitis with negative autoantibody, and 1 case as Bickerstaff’s brainstem encephalitis. The other 31 cases were not autoimmune encephalitis, including 14 cases of viral encephalitis, 2 case of purulent encephalitis, 2cases of tuberculous meningoencephalitis, 3 cases of central nervous system (CNS) tumor and 10 cases of epileptic disorders. All the autoantibodies except for Ma_2_ were positive in the CSF samples. All the patients exhibited acute or subacute onset. Symptoms are listed in Table 1.

**Table 1:** General clinical features

Characteristics	autoimmune encephalitis cases (*n* = 64)	non autoimmune encephalitis cases (*n* = 31)
Age of onset (yr)	Mean 36.24, Median 29 (14–76)	Mean 35.87, Median 39 (9–65)
Gender (M/F)	29/35	20/11
Course (d)	Mean 70.68, Median 27.5 (3–540)	Mean 51, Median 14 (1–360)
Fever (n)	29	13
Cognitive impair (n)	47	13
Memory deficits (n)	41	12
Seizure (n)	53	17
Psychiatric symptoms (n)	50	10
Movement disorder or dyskinesia (n)	26	3
Speech dysfunction (n)	23	10
central hypoventilation (n)	8	0
Autonomic dysfunction (n)	15	3
Decreased level of consciousness (n)	22	6
Ataxia (n)	1	0
Ophthalmoplegia (n)	1	1

### Diagnosis pathway of all cases following the diagnostic flowchart

**Fig. 1:**
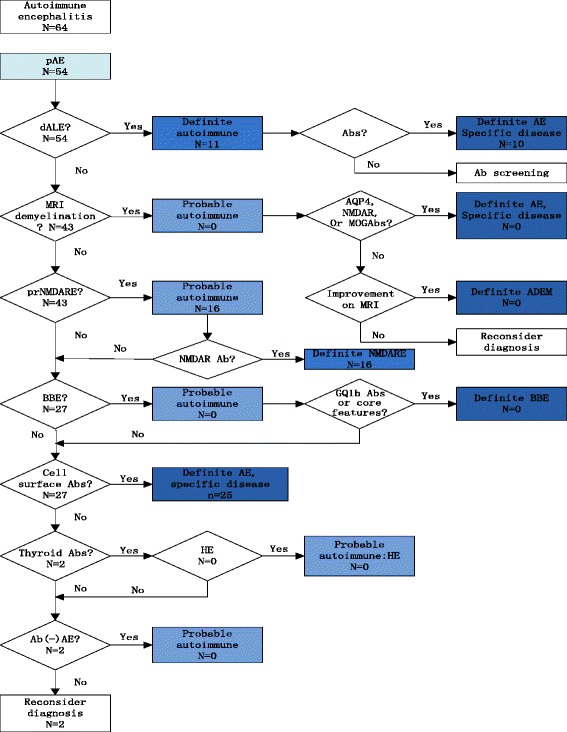
The diagnosis process of the 64 autoimmune encephalitis cases following the diagnostic flowchart. pAE: possible autoimmune encephalitis; dALE: definite autoimmune limbic encephalitis; prNMDARE: probable anti-NMDA receptor encephalitis; ADEM: acute disseminated encephalomyelitis; BBE: Bickerstaff’s brainstem encephalitis; HE: Hashimoto’s encephalopathy

**Fig. 2:**
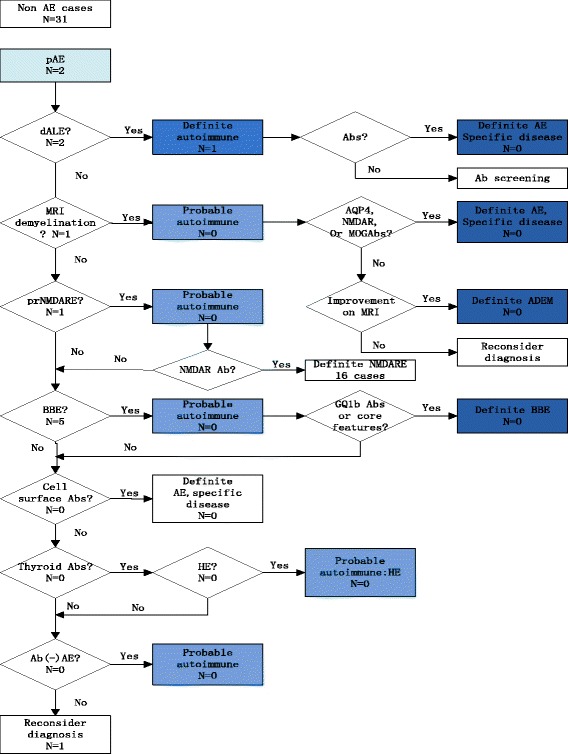
The diagnosis process of the 31 non autoimmune encephalitis cases following the diagnostic flowchart. pAE: possible autoimmune encephalitis; dALE: definite autoimmune limbic encephalitis; prNMDARE: probable anti-NMDA receptor encephalitis; ADEM: acute disseminated encephalomyelitis; BBE: Bickerstaff’s brainstem encephalitis; HE: Hashimoto’s encephalopathy

To evaluate diagnostic usefulness of the diagnostic flowchart as a whole, we list the diagnostic process in Fig. 1 and Fig. 2. Figure 1 shows the diagnosis process of the 64 autoimmune encephalitis cases following the flowchart. As we can see from the flowchart, 54 cases enter the flowchart. Eleven cases could be diagnosed as dALE, among which 10 cases need further antibody tests to determine their specific types. Among the remaining 43 cases, 16 cases were diagnosed as probable autoimmune encephalitis, the other 27 cases only can be diagnosed by antibody tests. Fig. 2 shows the pathway of the 31 non-autoimmune encephalitis cases in the flowchart. Two cases enter the flowchart, finally only 1 case was misdiagnosed as dALE, the other case ended the flowchart by reconsideration of the ‘pAE’ diagnosis.

### Diagnostic value of each criterion in the diagnostic approach

#### Criteria for ‘possible autoimmune encephalitis’ and ‘definite autoimmune limbic encephalitis’

With an assumption of unknown autoantibody status, 56 of the 95 patients (59%) met the criteria for ‘possible autoimmune encephalitis’ (pAE). Among the 64 autoimmune encephalitis cases, 54 (84%) cases were diagnosed as pAE. For the 31 cases of non-autoimmune encephalitis, 2 cases were misdiagnosed as pAE. Twelve of the 95 cases (13%) satisfied the criteria for ‘definite autoimmune limbic encephalitis’ (dALE). Eleven cases among the 64 autoimmune encephalitis patients were diagnosed as dALE. Moreover, for the 24 cases of limbic encephalitis, only 9 (38%) were diagnosed as dALE. For different autoantibody types, 35 of the 39 anti-NMDA receptor encephalitis cases met the pAE criteria, 2 cases met the dALE criteria. 11 of the 13 anti-LGI1 antibody-positive cases (85%) had a pAE diagnosis, and 7 (54%) cases were diagnosed as dALE. Among the 6 GABA_b_R-Ab (+) cases, 5 (83%) cases met the criteria for pAE, and only 1 (17%) case was diagnosed with dALE when autoantibody test results were unknown. Both paraneoplastic limbic encephalitis cases satisfied the criteria of pAE, while none met the criteria for dALE. AMPAR-Ab(+) limbic encephalitis met neither of the criteria for pAE or dALE, and the Ma2-Ab(+) limbic encephalitis could only be distinguished as pAE. One case of autoantibody-negative limbic encephalitis satisfied both criteria. Among the 31 non-autoimmune encephalitis cases, 2 cases were misdiagnosed as pAE and 1 case fulfilled the dALE criteria. Among the pAE (+) cases, 96% of the cases were true autoimmune encephalitis. Among the pAE (−) cases, 74% of the cases were not autoimmune encephalitis cases. The frequencies, sensitivities, specificities and accuracies are presented in Tables 2, 3 and 5.

**Table 2:** Frequencies of autoimmune encephalitis and ‘criteria for possible autoimmune encephalitis’

	Autoimmune encephalitis	Non autoimmune encephalitis cases
pAE(+)	54	2
pAE(−)	10	29

**Table 3:** Frequencies of autoimmune limbic encephalitis and ‘criteria for definite autoimmune limbic encephalitis’

	Autoimmune limbic encephalitis	Non limbic encephalitis
dALE(+)	9	3
dALE(−)	15	68

#### Criteria for ‘probable anti-NMDA receptor encephalitis’

Twenty (21%) of the 95 patients were eligible for ‘probable anti-NMDA receptor encephalitis’ (prNMDARE) diagnosis. Among the 39 cases of definite anti-NMDA receptor encephalitis, only 19 (49%) cases were diagnosed with prNMDARE with the assumption of unknown antibody status. The frequencies, sensitivities, specificities are presented in Tables 4 and 5.

**Table 4:** Frequencies of definite anti-NMDA receptor encephalitis and ‘criteria for probable anti-NMDA receptor encephalitis’

	Definite anti-NMDA receptor encephalitis	Non anti-NMDA receptor encephalitis
prNMDARE(+)	19	1
prNMDARE(−)	20	55

**Table 5:** Sensitivity, specificity and accuracy of the new diagnostic criteria

Criterion	pAE(%)	dALE(%)	prNMDARE(%)
Sensitivity	84	38	49
Specificity	94	96	98
Accuracy	87	81	78

#### Criteria for ‘possible autoimmune encephalitis’, ‘definite autoimmune limbic encephalitis’ and ‘probable anti-NMDARE’ in different disease courses

**Fig. 3:**
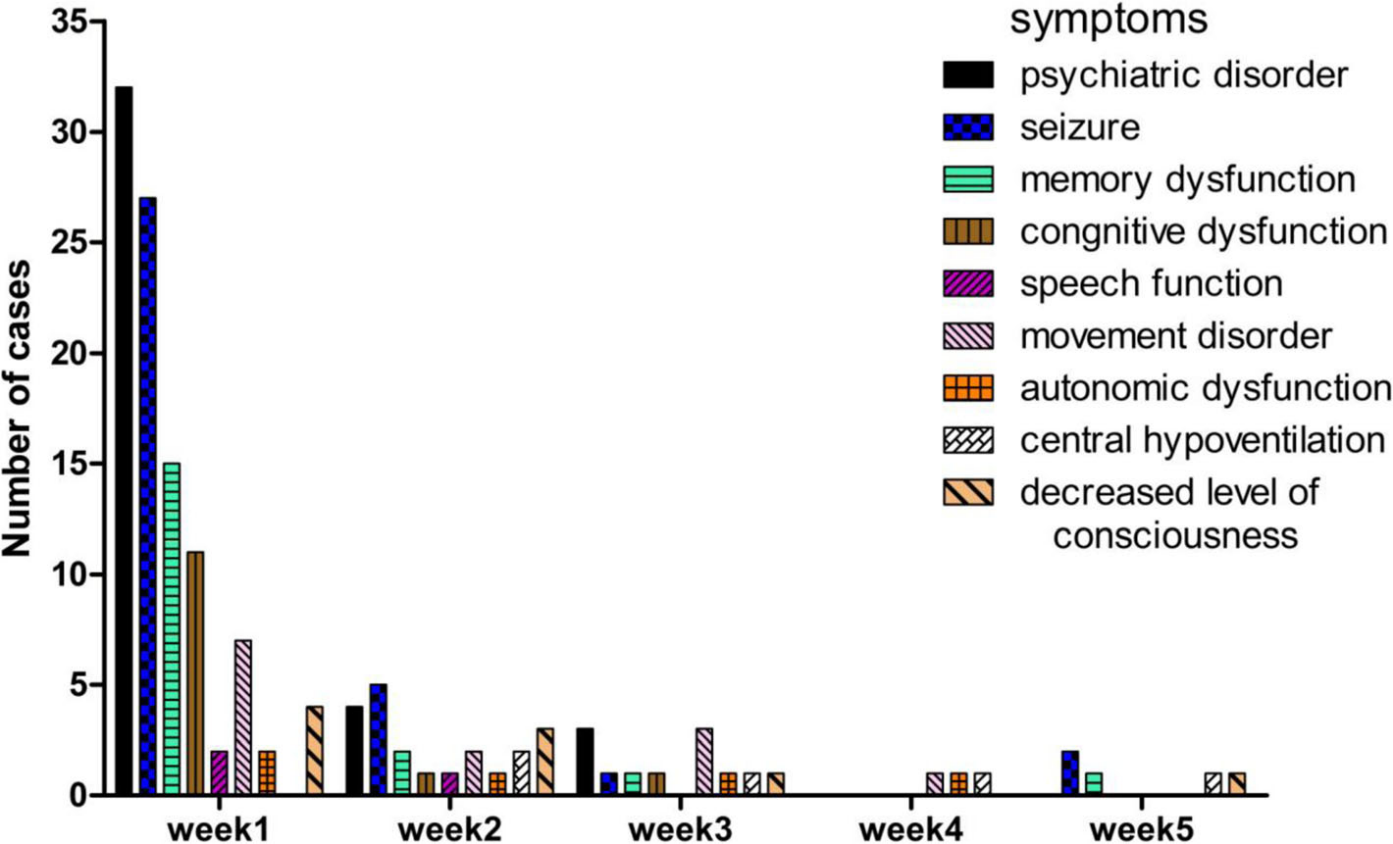
Number of cases when a symptom occurred for the first time in different periods. For cases ultimately-diagnosed as autoimmune encephalitis, most symptoms first appear in the first week except for central hypoventilation

**Fig. 4:**
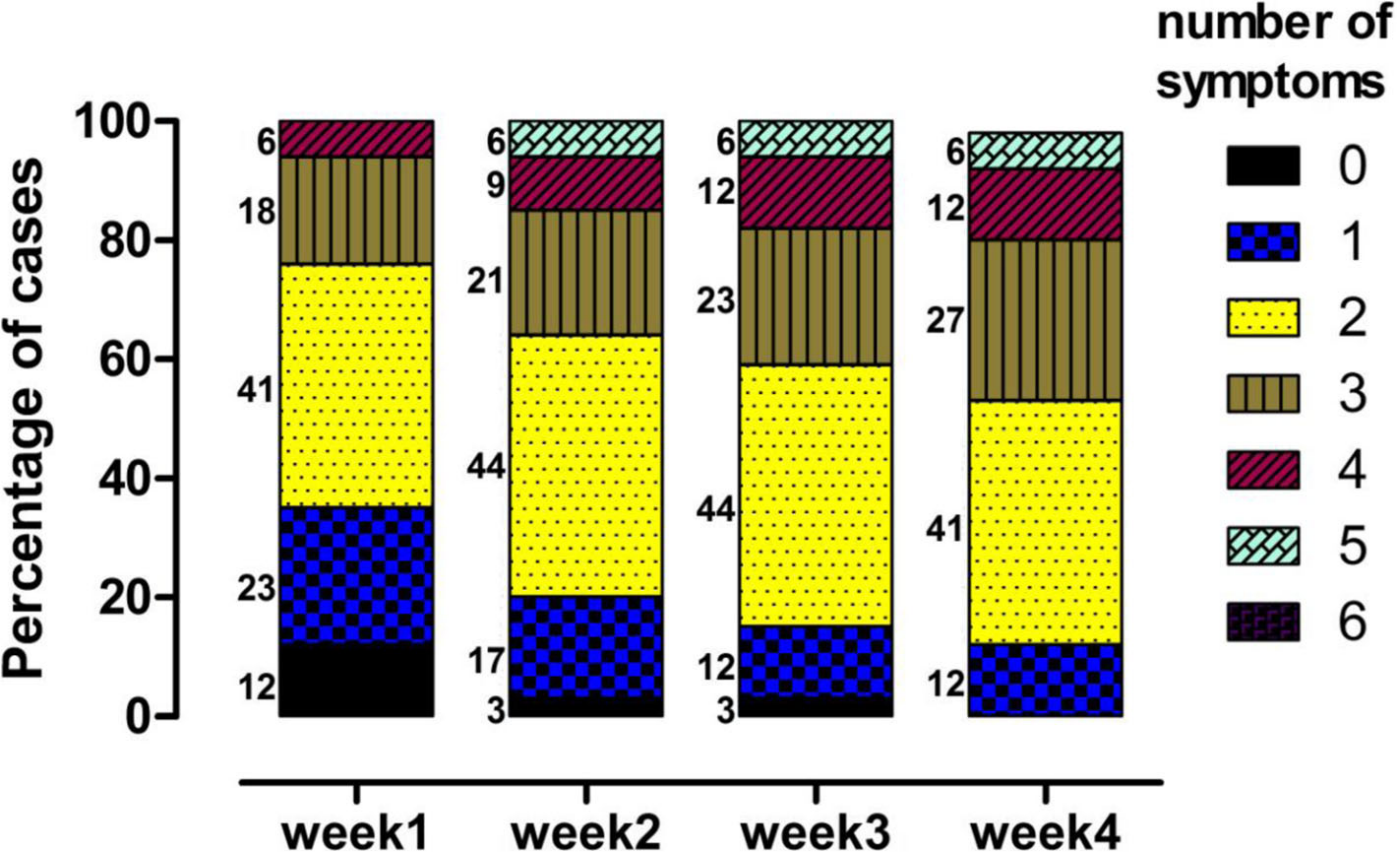
Percentage of confirmed anti-NMDAR encephalitis cases with different symptom numbers in different periods. Number of symptoms increased with time, however, more than 70% of cases were with less than 4 symptoms

Because autoimmune encephalitis typically has a subacute course, manifestations change with time. The number of cases when a symptom occurred for the first time in different periods in ultimately-diagnosed autoimmune encephalitis cases is summarized in Fig. 3. The most common symptoms during the first week were psychiatric symptoms, seizure, and memory impairment. Speech function and autonomic dysfunction account for a fraction. There is even no case with central hypoventilation. From the second week, the number of cases with central hypoventilation and autonomic dysfunction increased. Fig. 4 reveals that the number of symptoms of finally-diagnosed anti-NMDA receptor encephalitis cases increased with time, however, most cases presented with only 3 or less symptoms at the first four weeks. Even at the fourth week, merely 18% cases had 4 symptoms. Additionally, we calculated the sensitivity and specificity of pAE, dALE and prNMDARE criteria during four different periods (0-14 days, 15-30 days, 31-90 days, and >90 days) (Table 6, Table 7).

**Table 6:** Sensitivity of criteria for ‘possible autoimmune encephalitis’, ‘definite autoimmune limbic encephalitis’ and ‘probable anti-NMDA receptor encephalitis’

	0–14 days	15–30 days	31–90 days	>90 days	P*	
pAE^a^	60%(32/53)	70%(37/53)	74%(39/53)	81%(43/53)	0.123	
dALE^b^	10%(2/20)	15%(3/20)	15%(3/20)	35%(7/20)	0.184	
prNMDARE^c^	16%(5/31)	42%(13/31)	87%(27/31)	100%(31/31)	0.000	

^*^ *P* < 0.05 is significant

^a^sensitivity of possible autoimmune encephalitis assessed by pAE(+)/all the autoimmune encephalitis cases

^b^sensitivity of definite autoimmune limbic encephalitis assessed by dALE(+)/all the limbic encephalitis cases

^c^sensitivity of probable anti-NMDA receptor encephalitis assessed by prNMDARE(+)/all the anti-NMDAR encephalitis cases

**Table 7:** Specificity of criteria for ‘possible autoimmune encephalitis’, ‘definite autoimmune limbic encephalitis’ and ‘probable anti-NMDA receptor encephalitis’

	0–14 days	15–30 days	31–90 days	>90 days	P*
pAE^a^	83%(6/12)	92%(9/12)	92%(11/12)	92%(11/12)	0.880
dALE^b^	96%(43/45)	96%(43/45)	96%(43/45)	96%(43/45)	1.000
prNMDARE^c^	100%(34/34)	100%(34/34)	100%(34/34)	100%(34/34)	1.000

^*^ *P* < 0.05 is significant

^a^specificity of possible autoimmune encephalitis assessed by pAE(−)/non autoimmune encephalitis cases

^b^specificity of definite autoimmune limbic encephalitis assessed by dALE(−)/non autoimmune limbic encephalitis cases

^c^specificity of probable anti-NMDA receptor encephalitis assessed by prNMDARE(−)/non anti-NMDAR encephalitis cases

#### Criteria for ‘acute disseminated encephalomyelitis’, ‘Bickerstaff’s brainstem encephalitis’, ‘Hashimoto’s encephalopathy’ and ‘autoantibody-negative but probable autoimmune encephalitis’

None of the cases met the criteria for acute disseminated encephalomyelitis (ADEM), Bickerstaff’s brainstem encephalitis (BBE), Hashimoto’s encephalopathy (HE), or autoantibody-negative but probable autoimmune encephalitis. Among the 2 brainstem encephalitis cases (cases2 and 61), Case 2 did not satisfy the new BBE criteria because the patient had no decreased level of consciousness, even though its symptoms included mild external ophthalmoplegia, ataxia and the disappearance of the tendinous reflex. Furthermore, case 61 did not satisfy the criteria for the absence of ataxia, unconsciousness and external ophthalmoplegia.

## Discussion

An early and accurate diagnosis of autoimmune encephalitis is important to enable proper therapeutic interventions. Clinical manifestations of many diseases, including central nervous system (CNS) infections, septic/metabolic encephalopathy, drug toxicity, cerebrovascular disease and other disorders, resemble those of autoimmune encephalitis. Differentiation of these diseases relies not only on symptoms but also on accessory examinations. Detection of autoantibodies in the CSF and serum is particularly important for autoimmune encephalitis. The new approach provides criteria for the identification of autoimmune encephalitis regardless of antibody status. Data collected by our group provide an opportunity to investigate the frequency with which these patients were diagnosed with autoimmune encephalitis based on the new criteria and the diagnostic values of those criteria for possible and probable autoimmune encephalitis of different types.

### The value of the diagnostic flowchart as a whole

As a diagnosis approach, the flowchart is meant to use as a whole. On the one hand, the first diagnosis criteria is to screen as many autoimmune encephalitis as possible, thus high sensitivity is important for pAE criteria to reduce omission diagnostic rate. Once a patient enters the diagnosis flowchart, subsequent criteria contribute to distinguish false positive cases to lower misdiagnosis rate. On the other hand, as it shows in Fig. 1, 11 of the 54 pAE (+) cases could reach the ‘definite autoimmune’ level and 16 of the 54 pAE (+) cases reach ‘probable autoimmune’ level before antibody status is known. 27 (50%) cases can only be diagnosed by antibody tests. 10 of the 11 cases that are diagnosed as dALE also need antibody tests to determine their specific types. Because different autoantibodies may indicate different tumor types, furthermore, responses to treatment and long-term outcome seem to be different between LGI1 and anti-NMDA receptor encephalitis [11]. This may reveal the importance of antibody tests and the flowchart as a whole once again.

### Analysis of the diagnostic values for each criterion

The clinical manifestations of the autoimmune encephalitis cases that we summarized are consistent with previous publications [12, 13]. For diagnosis, assessing whether a suspicious case is caused by an autoimmune mechanism is important. Using the criteria for pAE, more than 80% of the autoimmune encephalitis cases were diagnosed as possible autoimmune encephalitis. The relatively high sensitivity of this approach offers clues to clinicians and enables exploration of autoimmune aetiologies. In our series, 10 cases did not meet pAE criteria. Because anti-virus antibodies were positive in 2 cases and the remaining 8 cases presented only one of the symptoms listed in the pAE criteria [11]. Reasons for the high specificity of pAE criteria are that MRI images of CNS tumors indicate difference from autoimmune encephalitis and symptoms of epileptic disorders are merely seizures. Viral encephalitis was the most difficult to be distinguished. Certain symptoms, such as psychiatric symptoms, are not specific to autoimmune encephalitis; in 2010, a study found no significant differences between autoimmune encephalitis and viral encephalitis groups [14]. Additionally, no differences were found in age, gender, or the presence of seizures, cognitive impairment, a confused state and headache between the two groups. Moreover, for diagnosis of viral encephalitis, aetiological tests are the golden standard, however, PCR or virus cultivation is uncommon in our clinic. What’s more, the positive rate of virus antibody tests is low and positive virus antibodies do not necessarily indicate a definite viral encephalitis diagnosis.

In the new approach, the authors proposed stricter criteria for specific diseases. For definite autoimmune limbic encephalitis, bilateral involvement of the medial temporal lobes on T2-weighted fluid-attenuated inversion recovery MRI was included in the dALE criteria. Most false negative cases did not meet this new criterion, resulting in low sensitivity. For anti-NMDA receptor encephalitis, a combination of common symptoms improved the diagnostic specificity. However, a large number of patients only presented with a few (less than four) symptoms in our series, resulting in low sensitivity.

From the above analysis, we propose that the new approach criteria for pAE have high sensitivity and provide an impetus to seek autoimmune aetiology, using specific criteria with high specificity for dALE, anti-NMDARE, BBE and ‘antibody-negative but probable autoimmune encephalitis’ could decrease the misdiagnosis rate. When clinicians refer to the new approach to make a diagnosis, we highly suggest that the approach be applied as a whole step-by-step procedure rather than selecting each criterion to use in isolation.

### Diagnostic values of the new approach in the early disease stage

Symptoms change as the disease progresses. Most patients develop decreased consciousness that progresses to a catatonic-like state, dyskinesia, choreoathetoid movements, dystonic posturing and abnormal ocular movements [15]. In anti-NMDA receptor encephalitis, 76% of patients have seizures during the first 3 weeks [10]. Therefore, we calculated frequencies of each symptom based on the time period. The most common symptoms in different time periods were consistent with a former case series based on the whole disease course [12], and most symptoms occurred during the first week. However, more than 70% of the patients presented with three or fewer symptoms during the early stage of the disease (the first two weeks), which resulted in low-level diagnosis (possible autoimmune encephalitis), particularly for definite autoimmune limbic encephalitis and anti-NMDA receptor encephalitis. The aim of the new clinical approach is early diagnosis and therapy; therefore, the above situation poses a challenge for clinicians trying to start immune-modulating therapy. Titulaer et al. reported in an anti-NMDA receptor encephalitis cohort study that 87% patients developed four or more symptoms in the first month [16]. Reason for the difference is that they categorized symptoms in 8 groups, among which ‘behavior and cognition’ and ‘memory’ merged into one item in the new approach, as well as ‘autonomic dysfunction’ and ‘central hypoventilation’. Thus the number of symptoms as diagnosis reference affects the chance of being included as prNMDARE.

We stratified the disease course into 4 groups. The sensitivity and specificity were consistent with the above results. The sensitivity of each specific criterion increases as the disease progresses. The specificity of pAE increases with time. The specificities of dALE and prNMDARE did not change with time because the two criteria ruled out confused cases by stricter standards. As we observed, the sensitivities of dALE and prNMDARE are very low in the first two weeks. Titulaer et al. reported that early immunotherapy correlated with good outcomes based on multivariate analysis of prognoses among 391 anti-NMDA receptor encephalitis cases [16]. Another study indicated that good clinical outcomes (defined by reductions in modified Rankin scores) were associated with early (<40 days) administration of immunotherapies in non-paraneoplastic patients [17]. However, in clinical practice, the advantage of the new approach in distinguishing autoimmune encephalitis may not emerge in the first two weeks, making it difficult for clinicians to start early immunotherapy. It is more reasonable to adopt criteria for specific types of autoimmune encephalitis with pAE criteria synthetically to avoid low sensitivity.

### Ability of the new approach to diagnose tumours

Paraneoplastic neurological syndromes (PNSs) were initially defined as neurological syndromes of unknown causes that were often associated with cancer [18, 19]. Broad syndromes associated with the nervous system are included. Among these syndromes, paraneoplastic encephalomyelitis and paraneoplastic limbic encephalitis present with symptoms of encephalitis. The discovery of antibodies against neural antigens expressed by the tumour (onconeural antibodies) has suggested that some PNSs are immune mediated [20, 21]. The new approach does not specifically put forward criteria for paraneoplastic encephalomyelitis and limbic encephalitis but instead indicates that dALE can be diagnosed if antibodies (Hu, Ma2, GAD, NMDAR, AMPAR, GABAbR, GABAaR, mGluR_5_, LGI1, CASPR_2_, and DPPX) are detected. Diagnosis of autoimmune encephalitis, which is based on positive autoantibody testing, contributes to tumour evidence. However, PNSs may occur in the absence of onconeural antibodies. Patients with tumours who are negative for autoantibodies may also be misdiagnosed. Ten paraneoplastic limbic encephalitis cases (judged by the PNS diagnostic criteria recommended by F Graus in 2004 [22]) published in the CNKI were collected and reassessed by the new approach. All the cases obtained a diagnosis of pAE (see Additional file 1), however, only 3 cases met the dALE standard. The PNS diagnostic criteria defined ‘classical syndromes’, ‘non-classical syndromes’, ‘well-characterized onconeuronal antibodies’, ‘partially characterized onconeuronal antibodies’ and combined neurological syndromes, onconeuronal antibodies and tumour evidence. The diagnosis of ‘definite paraneoplastic encephalitis’ did not thoroughly rely on the antibody level. Based on the complexity and diversity of PNSs, broadening the new approach to include PNSs is a reasonable and comprehensive goal.

Finally, some of the limitations of our study include the retrospective design and the possibility that the clinical data we acquired may be not completely direct and accurate. However, we collected cases with materials as detailed as possible, and considering the low incidence rate of the disease, a retrospective study is a relatively better choice. Besides, as the number of non-autoimmune encephalitis cases is low and non-autoimmune encephalitis group includes only a few diseases in our series, there may be bias with respect to ‘specificity’. Following this work, the new approach should be applied to prospective studies in the clinic.

## Conclusions

The new approach takes possible autoimmune encephalitis into account regardless of autoantibody status. The accuracy of every criterion is acceptable. When a case is diagnosed as possible autoimmune encephalitis with the new criteria, the chance of an accurate diagnosis is high. However, for autoimmune limbic encephalitis and anti-NMDA receptor encephalitis, criteria with low sensitivity and relatively high specificity may be more practical for excluding other diseases and reducing the misdiagnosis rate. Moreover, the sensitivity of pAE, dALE and prNMDARE are low in the first two weeks. For the individual patient, the number of symptoms was a limitation to obtain a probable or definite diagnosis at the early stage of the disease. It is difficult to distinguish autoimmune encephalitis patients and to perform immune-modulating therapy with only a ‘possible autoimmune encephalitis’ diagnosis at the early stage. The diagnostic criteria for PNSs were recommended for inclusion. For Chinese clinicians, this approach is restricted by the lack of state-of-the-art methods, such as pathogen detection. Furthermore, multi-centre prospective studies are greatly needed to acquire data on Chinese manifestations of the disease and therapy.

## Additional file

Additional file 1: Application of every criterion in the new approach in 10 paraneoplastic limbic encephalitis cases. 1 = YES, 2 = NO (XLSX 9 kb)

## Abbreviation

ADEM: acute disseminated encephalomyelitis
AMPAR: α-amino-3-hydroxy-5-methyl-4-isoxazolepropionic acid receptor
BBE: Bickerstaff’s brainstem encephalitis
CNKI: China National Knowledge Infrastructure
CNS: Central nervous system
dALE: Definite autoimmune limbic encephalitis
EEG: Electroencephalography
FN: False negatives
FP: False positives
GABA_b_R: Gamma-aminobutyric acid-B receptor
HE: Hashimoto’s encephalopathy
HSE: Herpes simplex encephalitis
LGI1: Leucine-rich, glioma inactivated 1
NMDA: N-methyl-D-aspartate
NSAbs: Neuronal surface antibodies
pAE: Possible autoimmune encephalitis
PNSs: Paraneoplastic syndromes
prNMDARE: Probable anti-NMDA receptor encephalitis
TN: True negatives
TP: True positives
